# Dual transcatheter edge-to-edge repair in a patient with cardiac amyloidosis and severe secondary mitral and tricuspid regurgitation: a case report

**DOI:** 10.1093/ehjcr/ytae601

**Published:** 2024-12-12

**Authors:** Julia Vogel, Peter Luedike, Amir Abbas Mahabadi, Tienush Rassaf, Lars Michel

**Affiliations:** Department of Cardiology and Vascular Medicine, West German Heart and Vascular Center, University Hospital Essen, Hufelandstr. 55, 45147 Essen, Germany; Department of Cardiology and Vascular Medicine, West German Heart and Vascular Center, University Hospital Essen, Hufelandstr. 55, 45147 Essen, Germany; Department of Cardiology and Intensive Care Medicine, Niels-Stensen-Kliniken, Marienhospital Osnabrück, Bischofsstr. 1, 49074 Osnabrück, Germany; Department of Cardiology and Vascular Medicine, West German Heart and Vascular Center, University Hospital Essen, Hufelandstr. 55, 45147 Essen, Germany; Department of Cardiology and Vascular Medicine, West German Heart and Vascular Center, University Hospital Essen, Hufelandstr. 55, 45147 Essen, Germany; Department of Cardiology and Vascular Medicine, West German Heart and Vascular Center, University Hospital Essen, Hufelandstr. 55, 45147 Essen, Germany

**Keywords:** Cardiac amyloidosis, Edge-to-edge repair, Cardiomyopathy, Mitral regurgitation, Tricuspid regurgitation, Case report

## Abstract

**Background:**

Mitral and tricuspid regurgitation in patients with cardiac amyloidosis (CA) pose significant diagnostic and therapeutic challenges due to its non-specific symptoms and limited treatment options. Transcatheter edge-to-edge repair (TEER) is complicated by altered cardiac geometry, advanced restriction, and potential amyloid valve deposits.

**Case summary:**

We present the case of dual TEER in a 79-year-old male with advanced transthyretin cardiac amyloidosis (ATTR-CA) and severe symptomatic mitral and tricuspid regurgitation. In a staged approach, TEER for both the mitral and tricuspid valves was successfully conducted, resulting in improved valvular function and symptom relief. Transvalvular gradients were 5 mmHg for mitral valve and 2 mmHg for tricuspid valve, each with mild residual regurgitation, improved clinical status, and regressive natriuretic peptides.

**Discussion:**

This case underscores the feasibility of dual TEER in CA patients with valvular involvement. Further research is necessary to optimize treatment strategies and address the multifaceted nature of this complex disease.

**Learning points**

- Feasibility of dual TEER in CA: This case demonstrates that transcatheter edge-to-edge repair (TEER) can be successfully performed on both the mitral and tricuspid valves in a patient with advanced cardiac amyloidosis (CA), highlighting a potential treatment avenue for those with high surgical risk and complex valvular pathology.
- Challenges and outcomes: Despite the inherent difficulties of performing TEER in CA patients due to factors such as global hypertrophy, small valve annulus, and thickened leaflets, the staged interventional approach resulted in improved valvular function, significant reduction in NT-proBNP levels, and enhanced patient symptoms and functional capacity, indicating promising outcomes for this patient cohort.

## Introduction

Cardiac amyloidosis (CA) is an infiltrative heart disease where cardiac deposits of amyloid fibrils lead to impaired function. The most common form of CA is transthyretin cardiac amyloidosis (ATTR-CA). Cardiac amyloid deposits affect the structure of the heart which leads to cardiomyopathy with progressive restriction complicated by conduction abnormalities and autonomic dysfunction.^1^ Biatrial enlargement promotes mitral and tricuspid valve regurgitation as a common comorbidity, further complicated by valvular amyloid deposits.^2^ Treatment is challenged due to intolerance to common heart failure medication and comorbidities, thus reciprocally worsening morbidity and mortality.^3^ Given this imminent medical need, the search for new treatment options remains a high priority. Transcatheter edge-to-edge repair (TEER) has emerged as a promising alternative to traditional surgery.^4^ Especially in multimorbid CA patients with elevated surgical risk, a catheter-guided procedure can be a favourable option.^5^ Transcatheter edge-to-edge repair has been previously documented in patients with ATTR-CA, but the distinct CA phenotype including global hypertrophy and restriction, small valve annulus, decreased stroke volume, thickened leaflets from amyloid deposition, and increased thromboembolic risk challenges this approach.^6^

We present the case of a 79-year-old male patient with advanced CA and severe mitral and tricuspid regurgitation receiving TEER of both the mitral valve (MV) and tricuspid valve (TV) as the first reported case in a single patient. This case highlights feasibility and delineates challenges of dual TEER in CA.

## Summary figure

Timeline from first examinations to follow-up examination. MV TEER and TV TEER refer to the values prior to intervention during the inpatient stay. hs-cTnI, high-sensitive cardiac troponin I; NYHA, New York Heart Association; NT-proBNP, N-terminal pro-brain natriuretic peptide; LVEF, left ventricular ejection fraction; MR, mitral regurgitation; MV, mitral valve; SV, stroke volume; TR, tricuspid regurgitation; TV, tricuspid valve.

**Figure:**
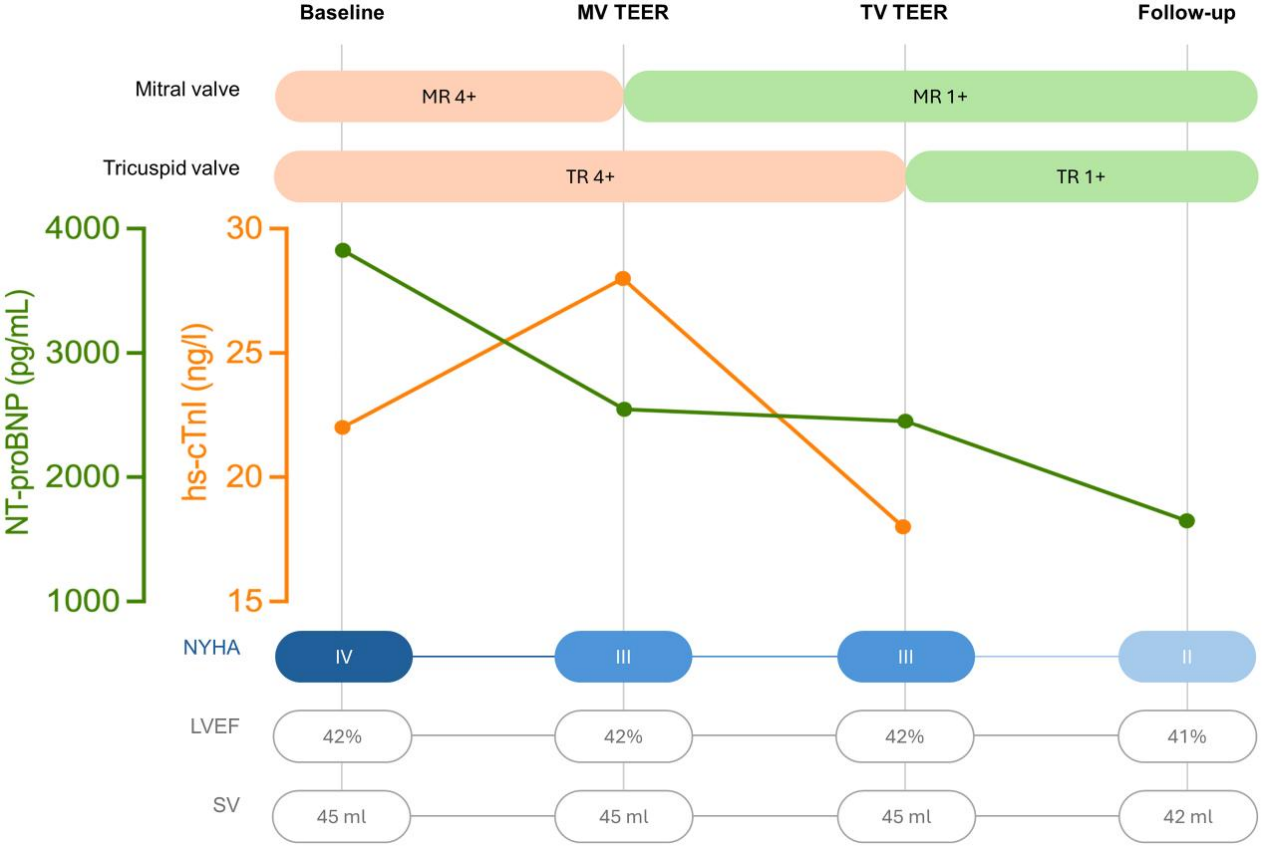


## Case presentation

The 79-year-old male patient was diagnosed with ATTR-CA in December 2022. He experienced persisting symptoms with dyspnoea New York Heart Association (NYHA) class IV despite optimized heart failure (HF) therapy, consisting of beta-blockers, MR antagonists, SGLT2 inhibitors, loop diuretics, and transthyretin-stabiliser therapy with tafamidis. The patient has a history of relevant comorbidities, including coronary artery disease and atrial fibrillation. Echocardiography revealed a concentrically hypertrophied ventricle and enlarged atria. The right ventricular function was mildly impaired, and right heart catheterization showed postcapillary pulmonary hypertension (mean pulmonary artery pressure 31 mmHg; pulmonary capillary wedge pressure 24 mmHg).

**Figure 1:**
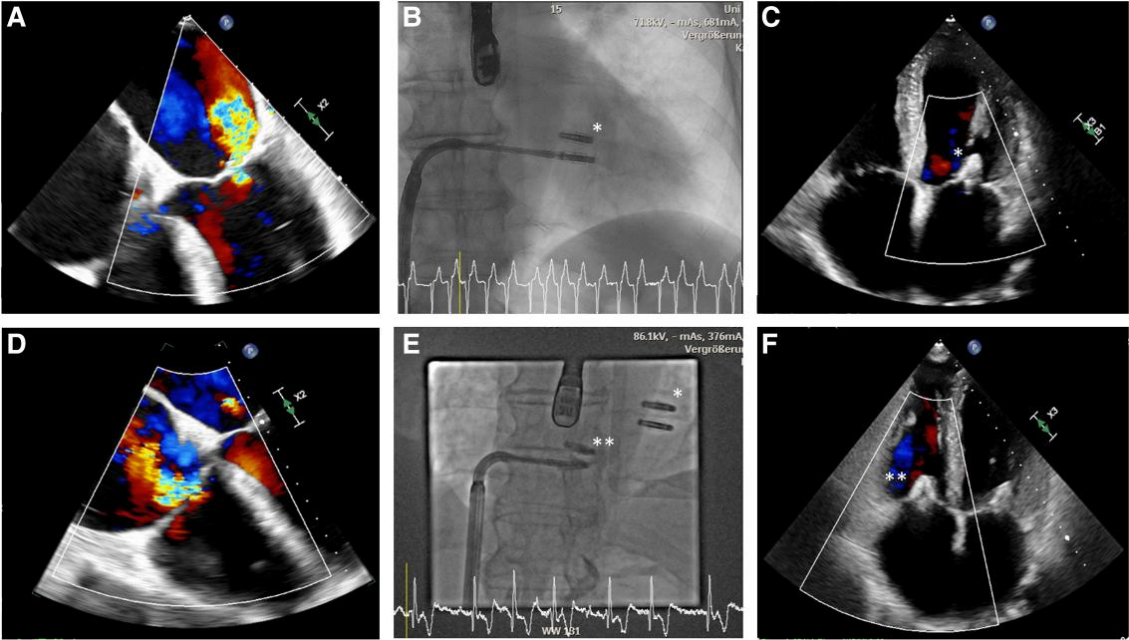
(*A*) TOE colour Doppler of MR before MV TEER. (*B*) TOE colour Doppler of TR before TV TEER. (*C*) AP fluoroscopic view as clips are inserted into MV. (*D*) TTE colour Doppler of MR after MV TEER. (*E*) AP fluoroscopic view as clips are inserted into TV. (*F*) TTE colour Doppler of TR after TV TEER. *Indicates PASCAL-Ace device in MV position. **Indicates PASCAL-Ace device in TV position. TOE, transoesophageal echocardiogram; TEER, transcatheter edge-to-edge repair; TTE, transthoracic echocardiography; AP, anterior–posterior; MV, mitral valve; TV, tricuspid valve; MR, mitral regurgitation; TR, tricuspid regurgitation.

The patient underwent further evaluation at the West German Heart and Vascular Centre, University Hospital Essen, Germany, which revealed severe MV and TV regurgitation on echocardiographic examination. Six-minute walk test was not possible due to resting dyspnoea, NT-proBNP was elevated with 3826 pg/mL (normal value < 125 pg/mL), and renal retention parameters were within normal range (*Summary figure*). Due to the patient’s hypotension and limited pharmacological options for HF management, a decision was made to proceed to interventional treatment. Progression of coronary disease was ruled out by coronary angiography. Transoesophageal echocardiogram (TOE) confirmed secondary mitral regurgitation (MR) with a vena contracta (VC) of 10 mm, an effective regurgitation orifice area (EROA) of 0.43 cm², and regurgitation volume of 64 mL (*Figure 1A*). Tricuspid regurgitation (TR) showed a VC of 9 mm, EROA was 0.69 cm^2^, and regurgitation volume was 67 mL (*Figure 1B*). Due to high surgical risk, the patient was planned for a staged interventional approach as per interdisciplinary heart team consensus. Low stroke volume (45 mL) and thickened valve leaflets were identified as intervention-specific risk factors that could complicate procedural success of TEER. The PASCAL-Ace (Edwards Lifescience, California, USA) devices were used for both procedures, which were performed with right femoral vein access with local anaesthesia and moderate sedation (Supplementary material online, *Video S1*).

The first intervention targeted the MV and was conducted in May 2023 with successful placement of two PASCAL-Ace devices (*Figure 1C*). A satisfactory result was achieved, with only first-degree mitral regurgitation and a post-interventional mean pressure gradient (MPG) of 3 mmHg (*Figure 1D*). No complications such as postoperative bleeding or rhythm disturbances were seen. No relevant shunt fraction from iatrogenic atrial septal defect after transseptal puncture was documented. Oral anticoagulation was continued the day following implantation. The patient was discharged on Day 6 following intervention. High-grade TR did not show relevant changes in follow-up.

In August 2023, the patient underwent the second interventional procedure to address high-grade TR with successful placement of two PASCAL-Ace devices (*Figure 1E*). Post-interventional echocardiographic examination showed a minimal residual TV insufficiency with a post-interventional MPG of 3 mmHg (*Figure 1F*). A duplex sonography of the puncture site showed an arteriovenous fistula with a flow volume of 200 mL/min which was considered non-haemodynamically relevant. No further therapy was necessary, and a follow-up 3 weeks later showed stable findings without an increase in flow volume. The patient was discharged 4 days post-tricuspid valve intervention.

The patient was closely monitored following both interventions, and a routine follow-up visit was scheduled three months after the last intervention. Here, the patient reported mild residual dyspnoea (NYHA II). Transthoracic echocardiography revealed an MV MPG of 5 mmHg and a TV MPG of 2 mmHg. LV function was quantified at 41%, with all devices in *loco typico*. Laboratory chemistry revealed a reduction in NT-pro BNP levels at 1649 pg/mL. The 6 min walk test showed a distance of 290 m. The post-interventional haematoma regressed, and the patient continued to report no pain or other related symptoms. The patient is currently (status May 2024) under routine follow-ups.

## Discussion

Cardiac amyloidosis presents significant challenges due to limited therapeutic options. When patients fail to respond sufficiently to medical therapy in presence of comorbidities as observed here, interventional approaches become necessary considerations. Despite the complexities posed by thickened valve leaflets, reduced stroke volume, and high surgical risk, TEER may represent a feasible option.

The outcomes of TEER in CA patients have generally been favourable, although success is influenced by the severity of amyloid infiltration, stroke volume, and overall cardiac function. Transcatheter edge-to-edge repair has been shown to reduce regurgitation and improve symptoms such as dyspnoea and exercise tolerance in many cases.^6^ Donà *et al*.^7^ demonstrated that patients with CA and MR have a higher likelihood of mortality compared to patients without CA. By addressing the valvular dysfunction, interventional therapies may help alleviate the burden of valve regurgitation, potentially reducing mortality and enhancing quality of life in patients with CA. Nevertheless, current studies on TEER of MR and TR in patients with CA are limited by small sample sizes, a lack of long-term outcome data, and mostly retrospective study designs. Additionally, the complex interaction between amyloid deposits and valvular structures remains insufficiently understood.^2^

**Figure 2:**
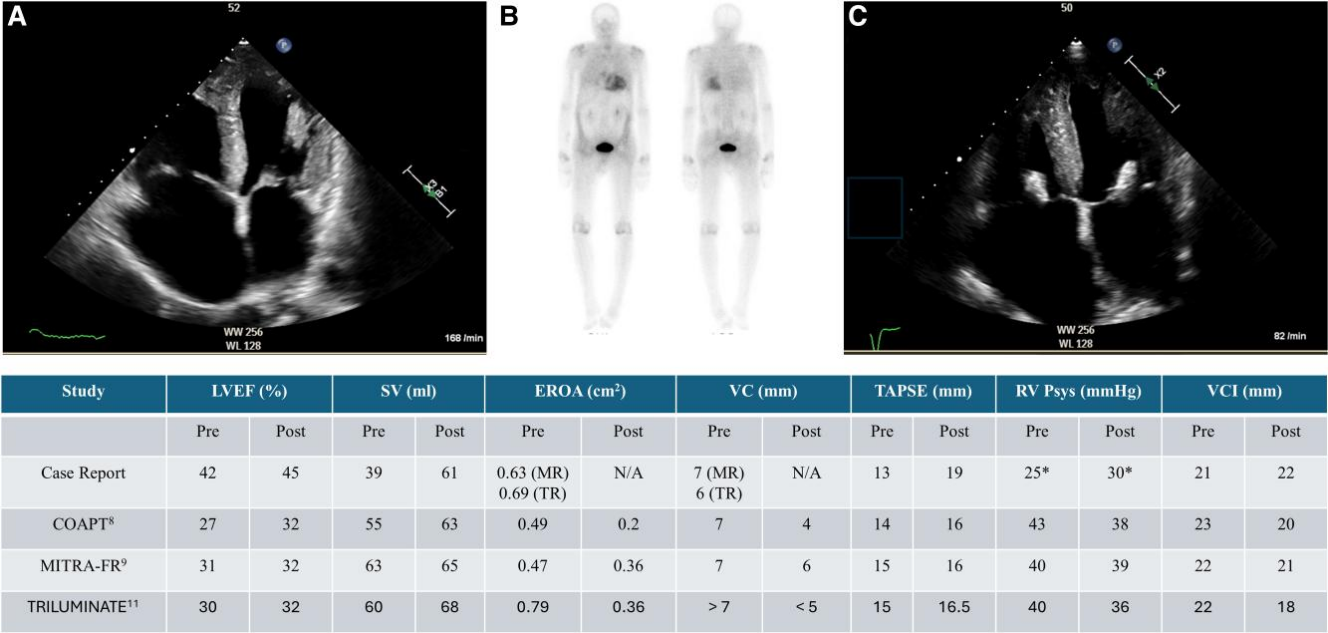
(*A*) TTE four-chamber view before MV and TV TEER. (*B*) Scintigraphy Perugini grade 2. (*C*) TTE four-chamber view after MV and TV TEER. EROA, effective regurgitant orifice area; LVEF, left ventricular ejection fraction; MV, mitral valve; MR, mitral regurgitation; SV, stroke volume; RV Psys, right ventricular systolic pressure; TEER, transcatheter edge-to-edge repair; TTE, transthoracic echocardiography; TV, tricuspid valve; TR, tricuspid regurgitation; VCI, vena contracta inferior; VC, vena contracta. *Potential underestimation due to concomitant severe TR. Data shown from Goel *et al*.,^8^ Obadia *et al*.,^9^ and Sorajja *et al*.^10^

In our patient, a staged interventional management of severe MR and TR led to notable improvements in valvular function and symptomatology. NT-proBNP significantly decreased, while the patient exhibited enhanced functional capacity in daily activities. We observed similar changes in the echocardiographic parameters compared to different studies in HF-patients without CA (*Figure 2*).^8–10^

Despite the initial beneficial effects of dual TEER on valvular function and symptom burden, long-term data on the outcomes of TEER in patients with CA are lacking. This includes potential effects on cardiac remodelling, recurrence of severe regurgitation, device-related stenosis due to progressive amyloid deposition, and late effects on symptom burden and performance status. The effects of novel specific therapies in ATTR-CA on valvular function are also unknown. Further prospective research on TEER in this specific patient population is needed.

## Lead author biography


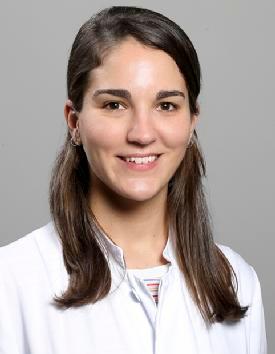
Dr Julia Vogel is a resident physician specialising in cardiology at the Department of Cardiology and Vascular Medicine, University Hospital Essen. Julia’s main interest is heart failure, particularly focusing on cardiac amyloidosis.

## Supplementary Material

ytae601_Supplementary_Data

## Data Availability

The data underlying this article cannot be shared publicly due to ethical reasons.

## Supplementary material

**Consent:** The authors confirm written consent for the submission and publication of this anonymized case report. The authors affirm their commitment to maintaining the highest standards of publication ethics in accordance with the guidelines established by the Committee on Publication Ethics (COPE) throughout the preparation and submission of this case report.

**Funding:** IFORES Clinician Scientist Scholarship from the Faculty of Medicine, University of Duisburg-Essen.
